# Polarized hyperspectral and polarized light microscopic imaging for enhanced visualization of white blood cells

**DOI:** 10.1117/1.JBO.31.4.046501

**Published:** 2026-04-15

**Authors:** Ximing Zhou, Hasan K. Mubarak, Ling Ma, Edward A. Medina, Bradley B. Brimhall, Marisa Whitted, Doreen Palsgrove, Madhu Shrestha, Baowei Fei

**Affiliations:** a University of Texas at Dallas, Center for Imaging and Surgical Innovation, Richardson, Texas, United States; b University of Texas at Dallas, Department of Bioengineering, Richardson, Texas, United States; c University of Texas Health Science Center at San Antonio, Department of Pathology and Laboratory Medicine, Texas, United States; d University of Texas Southwestern Medical Center, Department of Pathology, Dallas, Texas, United States; e Texas A&M University, Department of Diagnostic Science, School of Dentistry, Dallas, Texas, United States; f University of Texas Southwestern Medical Center, Department of Radiology, Dallas, Texas, United States

**Keywords:** polarized hyperspectral imaging (PHSI), polarized light imaging (PLI), Stokes vector, white blood cell

## Abstract

**Significance:**

White blood cells (WBC) are hematopoietic cells of the immune system that protect the body by recognizing and eliminating infectious agents. Abnormalities in WBC production, maturation, or function can lead to disease and associated morphologic changes that, when systematically characterized, support diagnostic classification and clinical decision-making.

**Aim:**

We aim to investigate polarized hyperspectral imaging (PHSI) and polarized light imaging (PLI) microscopy for the visualization of WBCs.

**Approach:**

We developed a dual-modality microscopic imaging system that performs both polarized hyperspectral imaging and polarized light imaging. In the dual imaging setup, we used a snapscan hyperspectral camera and an RGB camera to acquire images separately and further calculate four Stokes parameters (S0, S1, S2, and S3) as well as three Stokes vector-derived parameters, namely, the degree of polarization, degree of linear polarization, and degree of circular polarization. Synthetic RGB images of Stokes vectors and Stokes vector-derived parameters were generated for the visualization of cellular components with PHSI images. The spectral signatures of representative WBCs, e.g., granulocytes and lymphocytes, were extracted for qualitative comparison.

**Results:**

The preliminary results demonstrate that Stokes vector parameters can enhance the visualization of granules in granulocytes, the visualization of surface structures of lymphocytes, and the morphologic visualization of the monocyte nucleus. Furthermore, the results also reveal that the measured spectra of Stokes vector parameters could enhance the differentiation of WBCs in the spectral dimension, represented by the qualitative comparison between granulocytes and lymphocytes.

**Conclusions:**

Utilizing the spatial and spectral information from the Stokes vector data, our customized polarized hyperspectral microscopic imaging system enhances the visualization of WBCs and may provide a tool for the diagnosis of disorders related to white blood cells.

## 1 Introduction

White blood cells (WBCs) protect the body against foreign pathogens. They can be broadly classified into three different types: granulocytes, lymphocytes, and monocytes.^1^ Granulocytes are cells of the innate immune system that provide rapid, nonspecific, first-line defense and are distinguished morphologically by the presence of distinct cytoplasmic granules,^1^ which impart different functions in immune responses. Lymphocytes are central cells of the adaptive immune system that recognize specific antigens (e.g., molecules derived from pathogens or cancer cells) through specialized receptors. When a lymphocyte encounters a pathogen and binds the specific antigen that its antigen receptors recognize, it becomes activated, divides, and carries out effector functions such as antibody production by B cells or immune defense against infected or malignant cells by T cells.^2^^,^^3^ Monocytes are the largest type of WBCs and play critical roles in innate immunity, adaptive immune responses, and tissue repair. Differences in the number, proportion, and morphologic features (e.g., size, shape, granulation, nuclear size, and condensation) of WBCs are associated with a variety of diseases and conditions. For example, the presence of immature lymphocytes (i.e., lymphoblasts) or granulocytes (myeloblasts) in the peripheral blood in large numbers is a feature of acute leukemias.^4^ On the other hand, abnormally low concentrations of WBCs in the blood reduce the body’s defense against infections and may indicate that the bone marrow is failing to produce WBCs.

Flow cytometry is clinically useful for detecting immunophenotypic shifts or abnormalities in WBCs that may serve as indicators of infection, immune dysregulation, or malignancy. However, morphologic abnormalities in WBCs are not detected by flow cytometry; therefore, a manual blood smear examination using a microscope is necessary for comprehensive evaluation of circulating WBCs. Improving the tools used for a manual optical blood smear analysis can significantly enhance disease diagnosis. Optical imaging methods can provide complementary morphological, biochemical, and molecular-specific information about WBCs due to their advantages in resolution. Confocal microscopy,^5^ two-photon microscopy,^6^ third harmonic generation microscopy,^7^ Raman imaging,^8^ and fluorescence lifetime imaging^9^ are some examples that can be used to image WBCs. However, they all require expensive and complex optics, which makes it difficult to incorporate these techniques into the standard pathological imaging and routine clinical process.

Hyperspectral imaging (HSI) is a novel optical imaging technique, initially developed for remote sensing, and has evolved to find broad applications in numerous fields, including biology, medicine, and pathology applications.^10^

A three-dimensional (3D) hyperspectral data cube has two spatial dimensions and one spectral dimension with many discrete channels, allowing for more complex spectral analysis. Our group has actively investigated the usefulness of HSI in histopathology. We carried out head and neck squamous cell carcinoma (HNSCC) detection based on the morphology and spectral signatures of the nuclei. We found that both spatial and spectral information have a significant impact on the classification results.^11^ Using a deep neural network, we implemented whole-slide HNSCC detection and demonstrated that HSI outperforms conventional RGB imaging in several ways.^12^^,^^13^ Furthermore, by employing a pre-trained vision transformer on hyperspectral microscopic data, HSI has been applied to whole-slide images for thyroid cancer detection.^14^^,^^15^ With a fully automated hyperspectral imaging microscope being developed, HSI can be adapted into routine pathology analysis.^16^^,^^17^

Meanwhile, previous works have shown the usefulness of hyperspectral imaging for white blood cell classification. Li et al. used an acousto-optic tunable filter (AOTF)-based molecular hyperspectral system to distinguish the nucleus from the cytoplasm in WBCs.^18^ Robison et al. used a snapshot hyperspectral camera to differentiate red blood cells and WBCs on unstained slides.^19^ Duan et al. proposed a leukocyte (i.e., WBC) segmentation algorithm for automatic classification of WBCs using an AOTF-based hyperspectral system.^20^ Further research has combined HSI with deep learning methods to further improve classification accuracy.^21^^–^^24^

Polarized light imaging (PLI) acquires the polarization characteristics of light, which can reveal a wide range of structural features in biological tissues. There is significant research on the interaction of polarized light with biological tissue;^25^^,^^26^ however, few have investigated this interaction in WBCs. Current research largely integrates polarized light scattering with flow cytometry alone rather than other optical imaging to detect morphological differences in WBCs.^27^^,^^28^

Polarized hyperspectral imaging (PHSI) combines polarized light imaging with HSI to acquire the polarization state, spectrum, and spatial information of tissue samples. We are developing a dual-modality optical imaging microscope by combining hyperspectral imaging and polarized light imaging. We reported our preliminary study on the use of the PHSI microscope for distinguishing squamous cell carcinoma from normal tissue on hematoxylin and eosin (H&E)-stained slides based on the spectra of Stokes vector.^29^^,^^30^ We have also used PHSI combined with machine learning and deep learning to detect HNSCC on tissue slides.^31^^–^^33^ Our customized microscope can be easily incorporated into the standard pathological microscope for routine clinical analysis. In this study, we applied an RGB camera and an HSI camera to image white blood cells with polarized light imaging components to demonstrate that both PLI and HSI may complement each other as opposed to one or the other.

In this paper, we employ our customized dual-modality microscopic imaging system to enhance the visualization of WBC microstructures on Wright’s-stained blood smear slides. First, we describe how our customized microscope works in both PLI and PHSI modes. Then, we demonstrate the performance of our system in visualizing the granules in granulocytes, the nuclear morphologic features and surface structures of lymphocytes, and the nuclear morphologic features of monocytes. Finally, we show complementary spectral information in addition to the spatial information on Stokes vector parameters for differentiation of white blood cells. This study demonstrates that when combined, polarized hyperspectral imaging and polarized light imaging may have the potential to provide an imaging tool for improved visualization of white blood cells.

## 2 Methods

### 2.1 Pathology Slides

De-identified peripheral blood smear slides were prepared from blood samples submitted for routine clinical evaluation in the hospital pathology laboratory at the University of Texas Health San Antonio. Slides were either unstained or prepared with Wright’s stain. The research protocol was reviewed by the University of Texas Health San Antonio Institutional Review Board (IRB) (Protocol Number: HSC20200541N).

### 2.2 Polarized Hyperspectral Imaging & Polarized Light Imaging

**Fig. 1:**
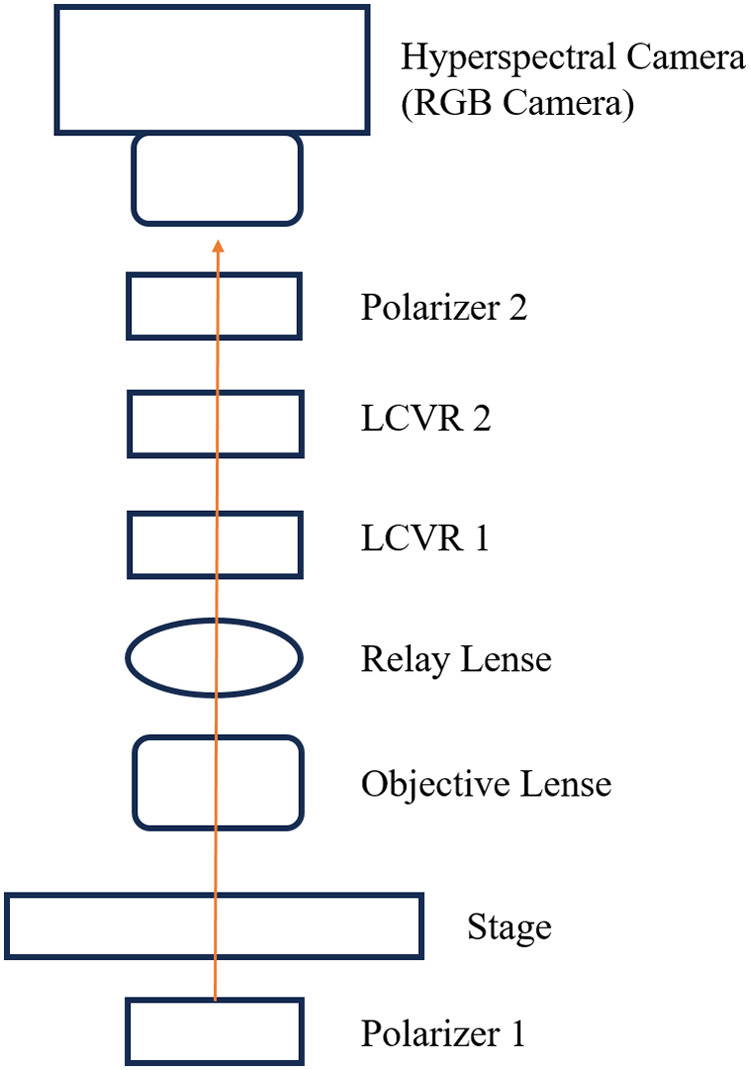
Setup of our customized polarized hyperspectral microscopic system.

Our customized microscopic imaging system acquires Stokes vector parameters under both the polarized light imaging setup and the polarized hyperspectral imaging setup, as we previously described.^34^ Stokes vector imaging is realized by two polarizers and two liquid crystal variable retarders (LCVRs). Figure 1 illustrates the setup of our customized microscopic system.

The transmissive axis of Polarizer 1 was set at 45 degrees, and the transmissive axis of Polarizer 2 was set at 0 degree. The fast axis of LCVR 1 was set at 0 degrees, and the fast axis of LCVR 2 was set at 45 degrees. The system is capable of full Stokes polarimetric imaging, which produces all four components of the Stokes vector. Thus, the system can completely define the polarization properties of the transmitted light. The way to calculate the four elements of the Stokes vector (S0, S1, S2, and S3) is expressed in Eq. (1):

$$S0=I_{h}+I_{v}\;S1=I_{h}-I_{v}\;S2=2*I_{45}-(I_{h}+I_{v})\;S3=2*I_{rc}-(I_{h}+I_{v})$$ (1)

where $I_{h}$ represents the light intensity measured with a horizontal linear analyzer, in which the retardations of LCVR 1 and LCVR 2 are both set at 0 rad; $I_{v}$ represents the light intensity measured with a vertical linear analyzer, in which LCVR 1 is set at 0 rad retardation and LCVR 2 is set at π rad retardation; $I_{45}$ represents the light intensity measured with a 45-degree oriented linear analyzer, in which LCVR 1 and LCVR 2 are both set at π/2  rad retardation; $I_{rc}$ represents the light intensity measured with a right circular analyzer, in which LCVR 1 is set at 0 rad retardation and LCVR 2 is set at π/2  rad retardation. The phase retardation of LCVR is determined by different values of voltage applied on it. In addition, the value of S0 is equal to the total light intensity.

After acquiring the four Stokes vector parameters, we calculated the Stokes vector-derived parameters, namely, the degree of polarization (DOP), the degree of linear polarization (DOLP), and the degree of circular polarization (DOCP), using Eq. (2):

$$\mathrm{DOP}=\sqrt{S1*S1+S2*S2+S3*S3}/S0\;\mathrm{DOLP}=\sqrt{S1*S1+S2*S2}/S0\;\mathrm{DOCP}=\sqrt{S3*S3}/S0$$ (2)

Compared with the polarized light imaging setup, the polarized hyperspectral imaging setup can help gain more insight into the interaction between white blood cells and light at different wavelengths by extracting the spectra of Stokes vector parameters. The polarized hyperspectral imaging setup of the microscopic system uses a snapscan hyperspectral camera instead of the RGB camera. In the polarized hyperspectral imaging dataset obtained by the system, each Stokes vector parameter corresponds to a 3D data cube with two spatial dimensions and one spectral dimension, as described in our recently published paper.^30^

### 2.3 Synthetic RGB Images

To generate synthetic RGB images from Stokes vector data cubes, we adopted an HSI-to-RGB transformation function similar to the spectral response of the human eye and modified it for our data.^11^^,^^12^ In the transformation process, three different spectral response curves are multiplied with the data cubes to generate three images at the three channels (red, green, and blue) of synthetic RGB images. We applied this HSI-to-RGB transformation function to all the Stokes vector-related parameters to generate PHSI-synthesized RGB images.

### 2.4 Spectra Extraction

To accurately extract the spectra of Stokes vector-related parameters from different types of white blood cells, we manually outlined the cell regions in the synthetic RGB images of S0 to generate the binary masks of cells. The cell masks generated from S0 were also applied to other Stokes vector-related parameters to extract the same regions of interest (ROIs) from the synthetic RGB images. In the next step, we applied the cell binary mask to the data cubes of Stokes vector-related parameters to extract the spectra of the cell ROI with mean from all the pixels in the ROI. This process helps reduce the influence of background pixels on the spectra of Stokes vector-related parameters.

## 3 Results

### 3.1 Polarized Light Images of Granulocytes

**Fig. 2:**
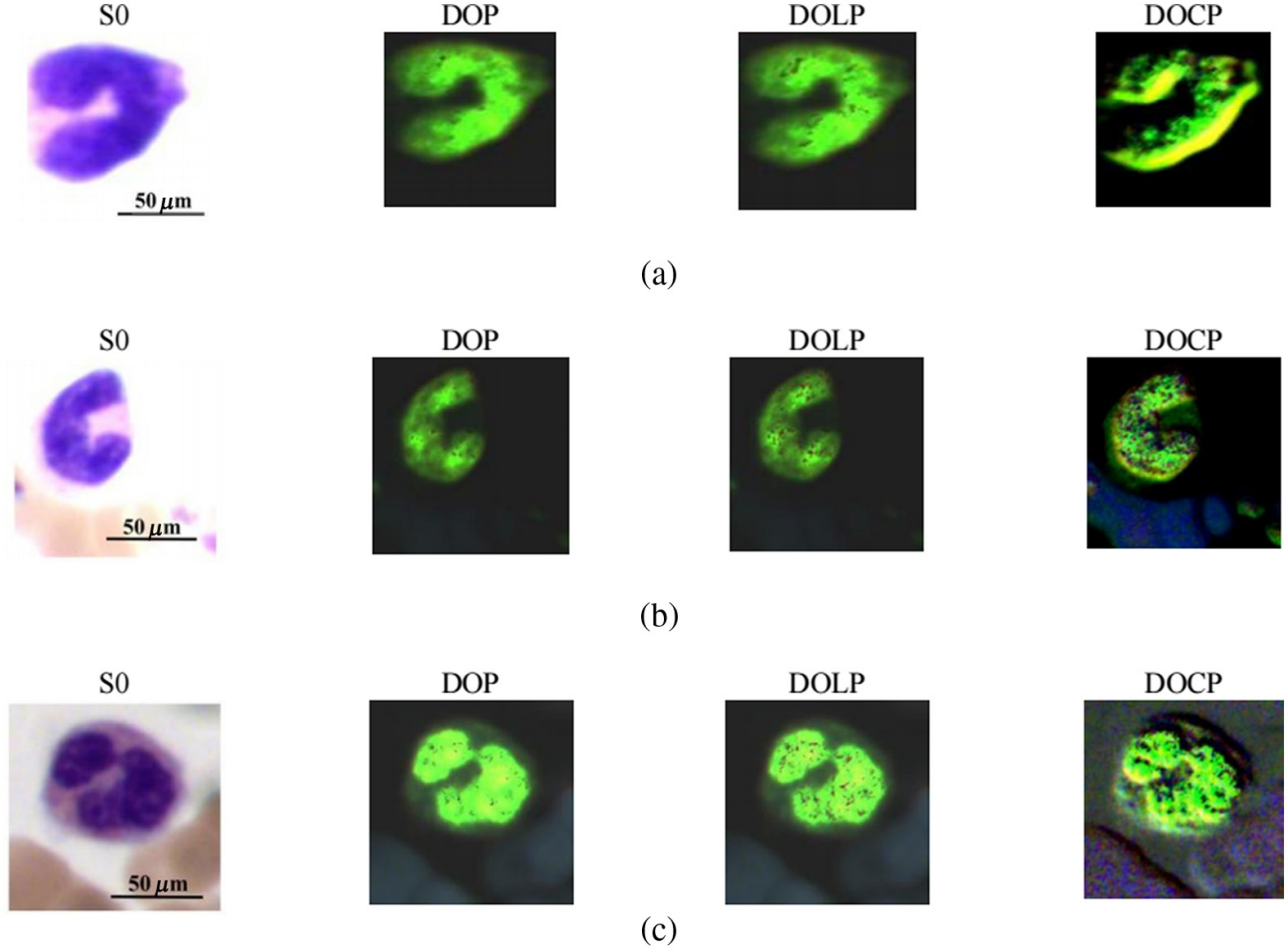
Synthetic RGB images of Stokes vector parameters S0, DOP, DOLP, and DOCP of (a) granulocyte 1, (b) granulocyte 2, and (c) granulocyte 3 on Wright’s-stained blood smear slides.

Compared with polarized hyperspectral imaging, polarized light imaging only requires an RGB camera rather than a hyperspectral camera, which is easier to implement. Therefore, polarized light imaging is a good choice if an application is only employed to obtain the spatial information of WBC. Figure 2 demonstrates the RGB images of Stokes parameter S0 and Stokes-derived parameters (DOP, DOLP, and DOCP) of three granulocytes, which we name as granulocyte 1, granulocyte 2, and granulocyte 3, from a Wright’s-stained blood smear slide. The preliminary results suggest that Stokes vector-derived parameters (DOP, DOLP, and DOCP) can improve the visualization of granules in granulocytes, and DOCP performs the best among the three parameters in visualizing granules. RGB images collected by polarized light imaging were used to generate the synthetic RGBs of S0, DOP, DOLP, and DOCP.

It can be seen that DOCP has the highest texture information with prominent nuclear surface details among these three granulocytes, which supports our claim that DOCP performs the best in visualizing granules.

### 3.2 Polarized Light Images of Lymphocytes

**Fig. 3:**
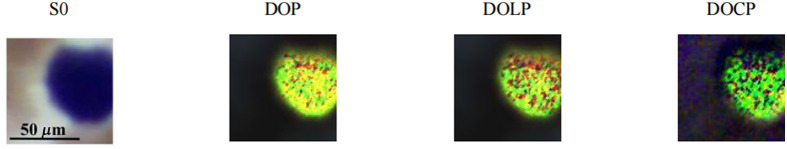
Synthetic RGB images of Stokes vector S0 as well as DOP, DOLP, and DOCP of a lymphocyte on a Wright’s-stained blood smear slide.

Fig. 3 demonstrates the RGB images of Stokes vector parameter S0, DOP, DOLP, and DOCP of a lymphocyte. The preliminary results reveal that Stokes vector-derived parameters (DOP, DOLP, and DOCP) improve the visualization of lymphocytes, and DOCP performs the best in visualization. Based on the information from the previously published paper,^2^ other light microscopic imaging techniques have been explored to image lymphocytes. The patterns we captured by our customized microscope on lymphocytes are similar to those captured by other types of microscopes, including epifluorescence microscopy, confocal microscopy, total internal reflection fluorescence (TIRF) microscopy, and two-photon laser scanning microscopy (TPLSM), which are featured with scattered dots and scattered lines. The surface patterns observed in the images may reflect differences in chromatin condensation and membrane organization and could, in part, be influenced by variations in surface protein density, such as antigen receptors, which differ by lymphocyte lineage, maturation (i.e., lymphoblast versus more mature), and activation state (i.e., exposed to antigen or not). There is a significant increase in the content of texture information in all images of S0, DOP, DOLP, and DOCP.

### 3.3 Polarized Light Images of Monocytes

**Fig. 4:**
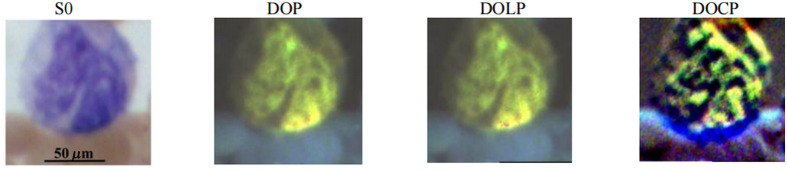
Synthetic RGB images of parameters S0, DOP, DOLP, and DOCP of a monocyte on a Wright’s-stained blood smear slide.

Figure 4 shows the RGB images of S0, DOP, DOLP, and DOCP of a monocyte. RGB images were collected by the polarized light imaging setup and used to generate synthetic RGBs of the parameters. From these images, the DOCP image shows the pattern of the nucleus.

The polarized light imaging setup could help visualize cellular components (granules, nuclear size, contour and degree of chromatin condensation, and membrane organization/cell surface structures) on granulocytes or lymphocytes and improve the image contrast of the large nucleus of monocytes. Although the visualization of monocytes by polarized light imaging did not show advantages in catching certain cellular components, such as granules, polarized light imaging could improve the image contrast of the monocyte nucleus.

### 3.4 Polarized Hyperspectral Imaging of WBCs

**Fig. 5:**
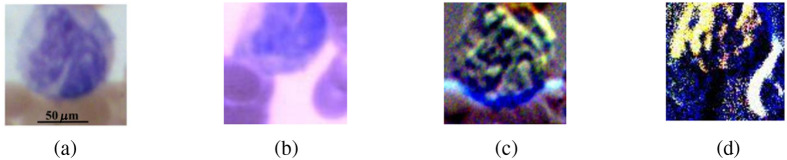
RGB images acquired from the polarized light imaging setup and synthetic RGB images collected from the polarized hyperspectral imaging setup of S0 and S3 from one monocyte. (a) The RGB image of S0 from the monocyte. (b) The synthetic RGB image of S0 from the monocyte. (c) The RGB image of S3 from the monocyte. (d) The synthetic RGB image of S3 from the monocyte.

In this study, we also employed the polarized hyperspectral imaging setup to collect spatial and spectral information simultaneously. Figure 5 demonstrates the synthetic RGB images of Stokes vector data cubes (S0 and S3) of one monocyte with the corresponding RGB images collected under the polarized light imaging setup. Figures 5(a) and 5(b) show the RGB image of S0 acquired from the polarized light imaging setup and the synthetic RGB image acquired from the polarized hyperspectral imaging setup of one monocyte. Figures 5(c) and 5(d) show the RGB image of S3 acquired from the polarized light imaging setup and the synthetic RGB image acquired from the polarized hyperspectral imaging setup of the same monocyte. From the representative images in Fig. 5, we find that the synthetic RGB images acquired under the polarized hyperspectral imaging setup and the RGB images acquired under the polarized light imaging setup achieve a similar image quality. To be specific, the two series of images both show an improvement in the visualization of the nucleus. The nuclear contours vary considerably (e.g., oval, kidney-shaped, horseshoe-shaped, irregular).

**Fig. 6:**
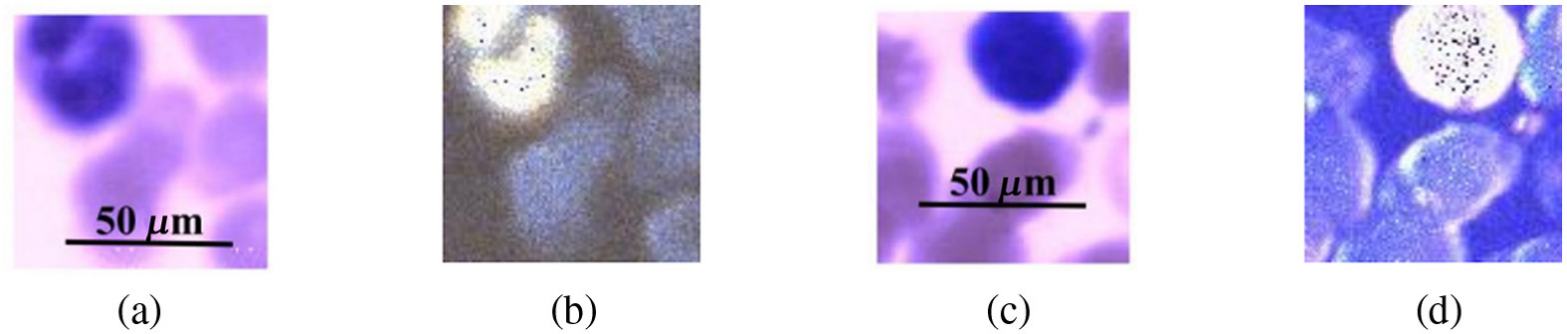
(a) The synthetic RGB image of S0 of one granulocyte, (b) the synthetic RGB image of DOP of the same granulocyte, (c) the synthetic RGB image of S0 of one lymphocyte, and (d) the synthetic RGB image of DOP of the same lymphocyte. All the images were acquired from a Wright’s-stained blood smear slide under the polarized hyperspectral imaging setup.

In this section, we prove that the synthetic RGB images from polarized hyperspectral imaging can improve the visualization of granules in granulocytes and nuclear surface structures of lymphocytes, as was shown for the polarized light imaging setup previously. Figure 6 shows the synthetic RGB images of S0 and DOP of one granulocyte and one lymphocyte.

From the representative images in Fig. 6, we can clearly observe the improved visualization of granulocyte and lymphocyte. To be specific, from the images of granulocyte, granules are hardly visible on S0 alone but can easily be seen on DOP. Similarly, more information on the surface structure of lymphocyte can hardly be observed on S0 but can easily be seen on DOP.

### 3.5 PHSI Spectra of Granulocytes and Lymphocytes

**Fig. 7:**
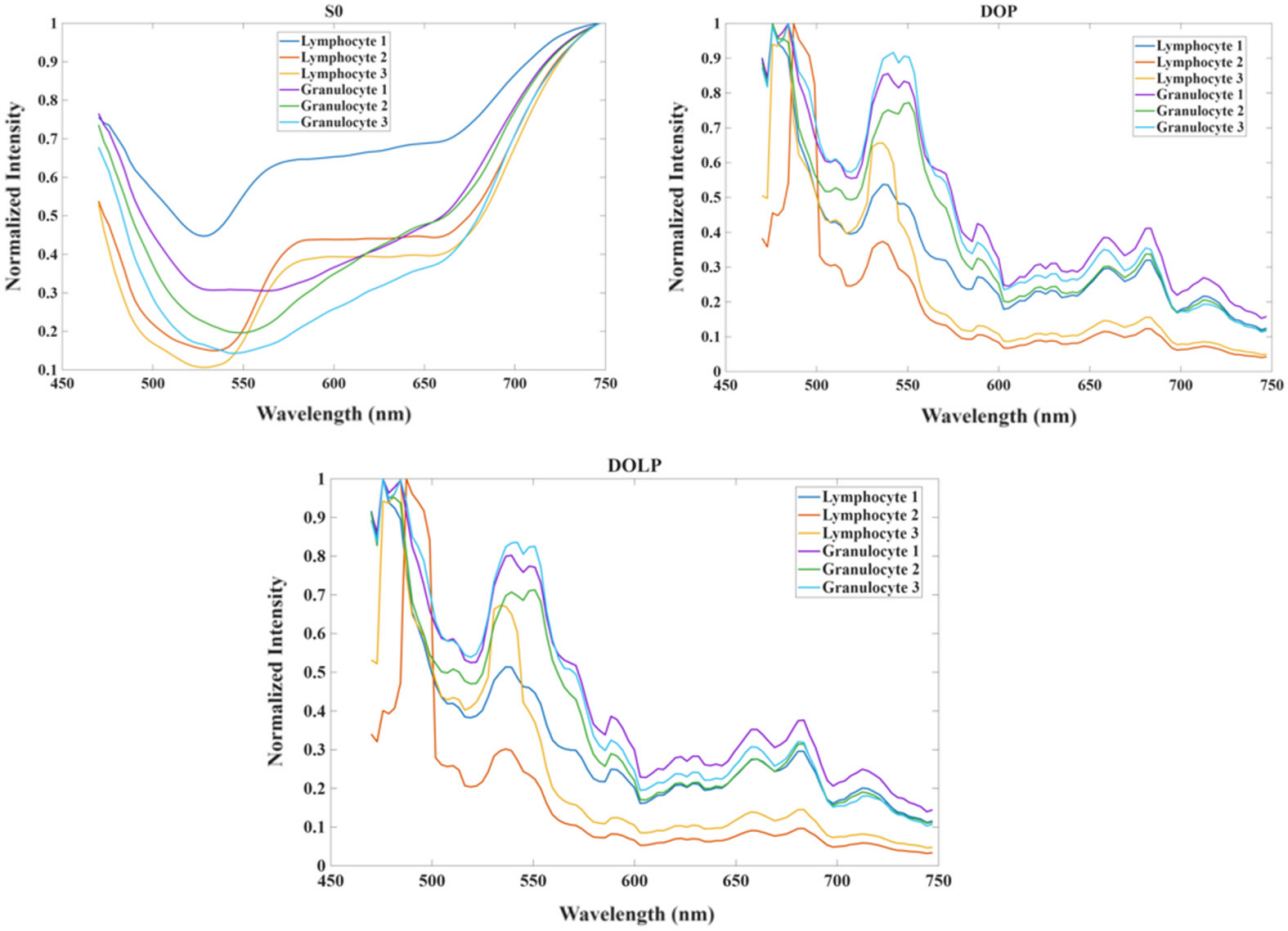
Mean spectra of S0, DOP, and DOLP of representative lymphocytes and granulocytes.

In addition to the structural information of white blood cells from the PHSI-synthetic RGB images, we could generate the spectra of Stokes vector-related parameters to analyze white blood cells in the spectral dimension. Figure 7 demonstrates the representative spectra of S0, DOP, and DOLP of three granulocytes and three lymphocytes. The spectra are generated based on the mean of all the pixels belonging to the cells. In our study, we observed not only the “low peak” at 525 nm but also peaks at other wavelengths, which is consistent with other literature: spectra curves of pathological slides usually have a “low peak” at the wavelength around 525 nm. The other peaks of spectra appear as a combined effect of hyperspectral and polarized light imaging. For S0, we found that the trends of the spectra from granulocytes and lymphocytes are consistent with each other (the normalized intensity decreases from 470 nm to 550 nm and then increases from 550 nm to 750 nm). For DOP, we could find that there are three peaks on both the spectra of granulocytes and lymphocytes. The first peak (high peak) of spectra appears near 475 nm. The second peak (low peak) of the spectra appears near 525 nm. The third peak (high peak) appears near 550 nm. For the DOLP, the spectra resemble those of DOP, with the three peaks appearing at similar positions. Regarding the differences of the spectra between granulocytes and lymphocytes, we can find that the spectra of granulocyte 1 and granulocyte 2 overlap largely with the spectra of lymphocyte 1, lymphocyte 2, and lymphocyte 3. However, there is much less overlapping on the spectra of DOP and DOLP between the spectra of three granulocytes and three lymphocytes, thus making it easier to differentiate the spectra.

**Fig. 8:**
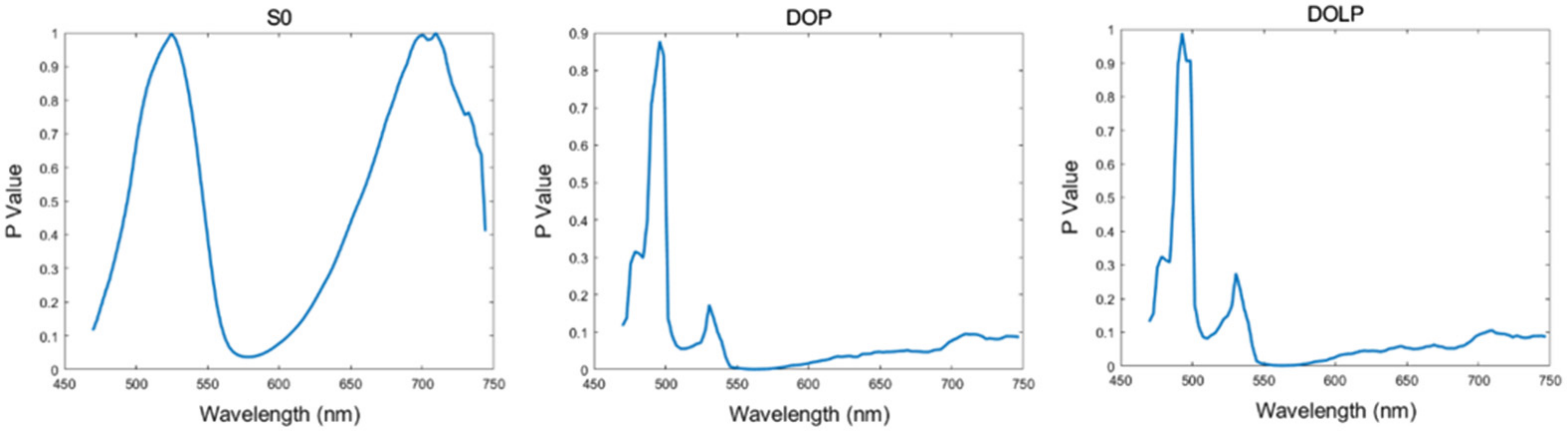
P values based on the two-sample t test among the mean spectra of 15 granulocytes and 15 lymphocytes.

Furthermore, we implemented a two-sample t-test on the mean spectra of S0, DOP, and DOLP of 15 granulocytes and 15 lymphocytes. The p values of different bands in each parameter are given in Fig. 8. It could be seen that the p values of DOP and DOLP significantly decrease compared with S0 at most wavelengths, and DOP and DOLP have the p values less than 0.05 at most wavelengths.

## 4 Discussion and Conclusion

In this study, we employed our customized microscopic imaging system to enhance the visualization of white blood cells on Wright’s-stained peripheral blood smear slides, with two different setups: polarized light imaging and polarized hyperspectral imaging. Our results suggest that our customized microscope with PHSI enhanced the visualization of granules in granulocytes. It can also enhance the visualization of morphologic features of lymphocytes, such as chromatin condensation and membrane organization/surface protein expression patterns (e.g., antigen receptors) that depend on the type of lymphocyte (B or T-cell), degree of maturation (i.e., lymphoblast versus more mature), and/or activation stages (i.e., exposed to antigen or not). Furthermore, it could enhance the visualization of monocyte nuclei. Our results also prove that our customized microscope is capable of revealing the spectral signatures of granulocytes and lymphocytes (the two most abundant white blood cells) based on Stokes vector and Stokes vector-derived parameters (S0, DOP, and DOLP). In addition, our customized microscope revealed its potential to better differentiate granulocytes and lymphocytes on peripheral blood smear slides by increasing the contrast between the spectra of granulocytes and lymphocytes. In addition, our results demonstrate that the spectra of different Stokes vector parameters (S0, DOP, and DOLP) have different performances in differentiating WBCs (represented by granulocytes and lymphocytes).

To the best of our knowledge, this is the first work to jointly apply polarized hyperspectral imaging and polarized light imaging to enhance the visualization of WBCs on Wright’s-stained peripheral blood smear slides. The preliminary study on WBC PHSI/PLI demonstrates that our customized microscope can enhance the visualization of WBCs and reveal the spectral signature of Stokes vector parameters of WBCs (e.g., granulocytes and lymphocytes). This study also applies PHSI and PLI to differentiate WBCs on Wright’s-stained peripheral blood smear slides. However, more work needs to be carried out on comparison with other microscopic imaging techniques and flow cytometry. By capturing both spectral and polarization-dependent information and by quantitatively analyzing Stokes vector parameter spectra of different types of WBCs, the polarized hyperspectral microscopic imaging technique may help study morphological characteristics in cell-cultured experiments and may enable analysis of cell–cell and cell–substrate interactions, cell surface characteristics, and dynamic changes in membrane-associated receptors and ligands. This technique may provide insights into cellular signaling, adhesion, and migration processes with high spatial localization without the need for exogenous dyes or destructive assays. In our future study, we plan to use our customized polarized hyperspectral microscope to image unstained samples, which will help to distinguish the difference between stained and unstained slides. The polarized hyperspectral imaging and polarized light imaging techniques can be further explored to study the diagnosis and prognosis of diseases and disorders of blood cells and various other cell types in the future.

## Data Availability

The datasets generated and analyzed during the current study are not publicly available.
